# Supplementary material for: Lessepsian migration and parasitism: richness, prevalence and intensity of parasites in the invasive fish Sphyraena chrysotaenia compared to its native congener Sphyraena sphyraena in Tunisian coastal waters
Source: PeerJ. 2018 Sep 14;6:e5558. doi: 10.7717/peerj.5558 (PMC6140674; doi:10.7717/peerj.5558)
Supplement: Table S1 [file peerj-06-5558-s001.docx]

| Parasites | Intermediate host | Definitive host |
| --- | --- | --- |
| *Lecithochirium fusiforme*  Luhe, 1901 | *Gibbula cineraria* | *Conger conger*  *Lophius piscatorius* |
| *Lecithochirium musculus* (Looss, 1907) | *Conger myriaster*  *Conger japonicus* | *Anguilla anguilla*  *Trachurus trachurus*  *Serranus hepatus*  *Crenilabrus cinereus*  *Conger conger*  *Atherina Hepsetus*  Serranus scriba |
| *Lecithochirium* *physcon*  Luhe, 1901 | - | *Lophius piscatorius* |
| *Lecithochirium rufoviride* (Rudolphi, 1819) | *Gibbula cineraria*  *Blennius pholis* | *Anguilla anguilla* |
| *Lecithochirium texanum* (Chandler, 1941) | - | *Euthynnus alleteratus* |
| *Lecithochirium jaffense* Fischthal, 1982 | *Seriola dumerili* | *Trachinotus ovatus*  *Epinephelus* sp.  *Echeneis naucrates*  *Pomatomus saltatrix*  *Gobius cobitus*  *Etrumeus golanii* |
| *Lecithochirium haifense* Fischthal, 1980 | - | *Atule djeddaba* |
| *Lecithochirium magnicaudatus*  (Fischthal & Kuntz, 1963) | - | Saurida undosquamis  *Labeo forskalii* |
| *Lecithochirium microstomum* Chandler, 1935 | - | *Trichiurus lepturus* |
| *Lecithochirium grandiporum* (Rudolphi,1819) | - | *Muraena helena*  *Lophius piscatorius*  *Conger conger*  *Saurida tumbil* |
